# Over-expression of survivin could prevent the oxidative stress and toxicity of rotenone in SH-SY5Y cells

**DOI:** 10.22038/IJBMS.2022.64345.14165

**Published:** 2022-07

**Authors:** Arman Rahimmi, Fardin Fathi, Bahram Nikkhoo, Farzad Soleimani, Mohammadbagher Khademerfan

**Affiliations:** 1 Department of Molecular Medicine, Faculty of Medicine, Kurdistan University of Medical Sciences, Sanandaj, Iran; 2 Cellular and Molecular Research Center, Research Institute for Health Development, Kurdistan University of Medical Sciences, Sanandaj, Iran; 3 Department of Anatomical Sciences, Faculty of Medicine, Kurdistan University of Medical Sciences, Sanandaj, Iran; 4 Liver and Digestive Research Center, Research Institute for Health Development, Kurdistan University of Medical Sciences, Sanandaj, Iran; 5 Department of Pathology, Faculty of Medicine, Kurdistan University of Medical Sciences, Sanandaj, Iran

**Keywords:** Apoptosis, Autophagy, Oxidative stress, Parkinson’s disease, Survivin

## Abstract

**Objective(s):**

It is important to find novel therapeutic molecular targets for curing Parkinson’s disease (PD). Accordingly, this study aimed to evaluate the effect of over-expression of the survivin gene, a gene frequently reported as neuroprotective, on the *in vitro* model of PD.

**Materials and Methods:**

Survivin was over-expressed in SH-SY5Y cells. Next, the cells were treated with rotenone (500 nM) for 24 hr. Then, viability and the total antioxidant capacity were assessed. The expression levels of 15 important genes of key cellular processes (oxidative stress, apoptosis, cell cycle, and autophagy) were assessed. The studied genes included survivin, superoxide dismutase, catalase, BAX, bcl2, caspase 3, caspase 8, caspase 9, p53, SMAC, β-catenin, mTOR, AMPK, ATG7, RPS18. The apoptosis level and the frequency of cell cycle stages were assessed by flow cytometry. For analyzing the data, the ANOVA test followed by Tukey’s test was used to evaluate the significant differences between the experimental groups. *P*<0.05 was considered significant.

**Results:**

Survivin could significantly decrease the rotenone-induced apoptosis in SH-SY5Y cells. The rotenone treatment led to down-regulation of catalase and up-regulation of bax, bcl2, caspase 3, caspase 8, P53, β-catenin, and ATG7. Survivin could significantly neutralize the effect of rotenone in most the genes. It could also increase the total antioxidant capacity of SH-SY5Y cells.

**Conclusion:**

Survivin could prevent the toxic effect of rotenone on SH-SY5Y cells during the development of *in vitro* PD model via regulating the genes of key cellular processes, including anti-oxidation, apoptosis, cell cycle, and autophagy.

## Introduction

Parkinson’s disease (PD) is the second most common neurodegenerative disease, which is developed due to significant loss of dopaminergic neurons in the nigrostriatal pathway in the brain. The symptoms of PD include movement disorders (e.g., resting tremor, rigidity, bradykinesia) and non-movement disorders (e.g., cognition impairments and dementia) (1). The prevalence of PD is estimated to be 10 million cases worldwide, with one million new cases annually (2, 3). The prevalence of the disease has increased by 74% from 1990 to 2016 and is still increasing. It is important to know that the increase was not solely due to the increase of the elderly people in the world population (22%). It is argued that the increase in PD prevalence may be due to several factors, such as changes in the environment and human lifestyle (2). The social and economic burden of PD is enormous such that the life quality of the patients fall severely and several billion dollars are spent on medical care and other indirect costs annually only in the US (3). 

Currently, there is no definite cure for PD. The common medications used for PD (e.g., levodopa, pramipexole, amantadine, and selegiline) usually increase the level of dopamine in the brain. However, the medications only alleviate and manage the symptoms of PD, without inhibiting or attenuating the gradual loss of dopaminergic neurons. The medications have also serious side effects in long-term use. Accordingly, developing novel medications and therapeutic approaches, which alleviate the symptoms of PD and attenuate the progressive loss of dopaminergic neurons, has a great value. 

As mentioned above, the death of dopaminergic neurons within the nigrostriatal pathway mainly occurs via apoptosis (4). Thus, inhibition of the apoptosis pathway in the cells may have a neuroprotective role and prevent their loss. This idea is supported by many studies (5). Therefore, it is probably possible to prevent the development/progression of PD by inducing anti-apoptotic pathways and over-expressing the related genes in dopaminergic neurons. The inhibitors of apoptosis proteins (IAPs) are an important family of proteins, which bind to the caspases and prevent their activation (6). Survivin is a member of the IAPs family with a molecular weight of 16.5 kDa. Survivin is encoded by “baculoviral inhibitor of apoptosis repeat-containing 5” (BIRC5) gene, which is placed on the chromosomal location 17q25.3. Previous studies showed that survivin prevents activation of caspase 3 and caspase 7 (7). However, “second mitochondria-derived activator of caspases” (SMAC) can inhibit survivin and prevent survivin’s inhibitory function on the caspases (8). Additionally, survivin can help the progression of the cell cycle by inhibiting p53. This dual role of survivin makes it distinguished from other IAPs (7). It is also noteworthy that survivin is a target of the Wnt/ β-catenin pathway, in which the activated β-catenin increases the expression level of survivin (9). 

The down-regulation of survivin was reported in some of the animal models of neurodegenerative diseases (e.g., Amyotrophic lateral sclerosis (ALS) and brain stroke) (10, 11). Accordingly, it was hypothesized that reinforcing the signal may aid in the treatment of neurodegenerative and cognitive impairments (12). Various studies proved the neuroprotective effects of survivin. In this regard, Sehara *et al*. (2013) showed that survivin was a target for STAT3. So, the increase in the expression level of each one of the genes was associated with neuroprotective effects on the hippocampus of the mouse brain-stroke model (13). Additionally, Baratchi *et al*. (2010 and 2011) did extensive studies on the roles of survivin in neural cells. They concluded that survivin may be an interesting therapeutic target since it makes a wide regulatory network with several types of proteins in certain cellular pathways (e.g., apoptosis, oxidative stress, and cell cycle) to imply its neuroprotective features (7, 14). A study (2020) showed that inhibition of survivin led to an increase in oxidative stress level, a decrease in superoxide dismutase (SOD) gene level, and consequently increase in the mortality of cancer stem cells in breast tumors (15). It seems that the autophagy process is another target of survivin. In this regard, Roca *et al*. (2008) reported that the increase in the expression level of mTOR (a key inhibitor of the autophagy process) led to an increase in the expression level of the survivin gene (16). However, to the best of our knowledge, no study has been done yet on the protective effects of wild-type survivin on *in vitro* and *in vivo* models of PD. Additionally, it is worthy to evaluate the probable effects of survivin on the models via analyzing the genes involved in apoptosis, oxidative stress, and autophagy pathways. 

According to the above explanations, this study aimed to evaluate the neuroprotective effect of over-expression of the survivin gene on the* in vitro* model of PD.

## Materials and Methods

***Cell culture***

SH-SY5Y cell line was supplied from the Pasteur Institute of Iran (Tehran) and it was authenticated using short tandem repeat analysis, which confirmed the genotypes at all examined loci. The cell line was cultured in DMEM/F12 medium (Invitrogen), supplemented with 10% fetal bovine serum (FBS) (Invitrogen), 1% GlutaMax (Invitrogen), and 1% penicillin/streptomycin (Invitrogen). The cells were kept in the incubator (Memmert) under an atmosphere with 5% CO_2_, 90% humidity, and 37 °C temperature. The culture medium was replaced every 48 hr. Passaging of the cells was performed by using trypsin-EDTA 0.25% (Invitrogen) in phosphate-buffered saline (PBS). The cultures were incubated for 48 hr for attachment and reached 70% confluence before the treatments. This condition was the same for all normal and genetically manipulated groups in this study.

***Overexpressing the survivin gene in SH-SY5Y cell line***

Firstly, the coding sequence (CDS) of survivin was obtained from the NCBI database (CCDS32751.1). Then, other necessary sequences, including restriction endonuclease cleavage sites (EcoRI recognition site at upstream and BamHI recognition site downstream of the CDS) and Kozak sequence (GCCGCCATG at upstream of the CDS) were added to appropriate upstream/downstream positions of the survivin CDS using SnapGene software version 3.2.1. Next, the final sequence was synthesized (Pishgam Biotech Co, Iran) and inserted into the pCDH expression vector (pCDH-CMV-MCS-EF1-CopGFP-T2A-Puro) (Bonyakhteh, Iran). The final vector was cloned in *Escherichia coli* *DH5α*. After amplification, the cloned vector was extracted (Qiagen Plasmid Mini Kit, United States) and transfected into SH-SY5Y cells using lipofectamine 2000 (Thermo Fisher, United States). The appropriate insertion of the survivin sequence and the successful cloning of the final vector in *E. coli* were confirmed by Sanger sequencing and colony PCR methods. Also, the efficiency of the vector transfection into SH-SY5Y cells was confirmed by dividing the number of green cells under a fluorescent microscope (GFP-positive cells) into all cells. 

***Microscopic imaging conditions***

The medium of SH-SY5Y cells (with 70% confluence) was discarded, washed twice with PBS, fixed with paraformaldehyde (5%), washed with PBS twice, and incubated with 4′,6-diamidino-2-phenylindole (DAPI) 0.2 μg/ml dissolved in PBS. Next, the cells were washed twice and observed under a microscope with a 20x objective lens (model IX71 Inverted Fluorescence Microscope, Olympus, Japan) in a dark room to minimize the light noises. 

First, the cells were imaged with a U-MWB2 filter (blue light) to observe green-colored cells (transfected cells). Next, the cells were imaged with a U-MWU2 filter (UV light) to observe all the cells (stained with DAPI). Then, the two images were merged using Image J software. 

***Experimental groups and treatments***

Seven different treatments were applied on normal or genetically manipulated SH-SY5Y cells (70% confluence) as follows: 

I. Normal SH-SY5Y cells were kept for 24 hr as described in section 2.1 (Healthy normal group). 

II. Normal SH-SY5Y cells were treated with rotenone (500 nM) for 24 hr. 

III. Genetically manipulated (Overexpressing survivin gene) SH-SY5Y cells were kept for 24 hr as described in section 2.1. 

IV. Genetically manipulated (Overexpressing survivin gene) SH-SY5Y cells were treated with rotenone (500 nM) for 24 hr. 

V. Genetically manipulated (null vector) SH-SY5Y cells were kept for 24 hr as described in section 2.1. 

VI. Genetically manipulated (null vector) SH-SY5Y cells were treated with rotenone (500 nM) for 24 hr. 

VII. Normal SH-SY5Y cells were treated with rotenone vehicle (DMSO) for 24 hr.

***Randomizing the treatments and blinding the experimental groups to the researchers***

In each experiment, a researcher selected the culture flasks/plates/wells randomly for the treatments and marked them with codes. Then, another researcher, who was blinded to the treatment groups, performed the assessments and reported them to the third researcher (who was also blinded to the treatment groups) for statistical analysis. Finally, the first researcher received the results of the assessments and assigned the codes to their respective treatment groups in the presence of other researchers. 

***MTT assay***

The cells were cultured in 96-well plates until their confluence reached 70%. Then, they went under the treatments for 24 hr. Next, the media was discarded from cell cultures. 50 µl of serum-free media and 50 µl of MTT solution (5 mg/ml solution in PBS) were added to each well. The plate was incubated at 37°C for 3 hr. After incubation, 150 µl of MTT solvent (4 mM HCl, 0.1% NP-40 in isopropanol) was added to each well. The plate was placed in the dark and shook for 15 min to dissolve the MTT formazan. Finally, the absorbance was read at 590 nm using a Synergy™ HTX Multi-Mode Microplate Reader (BioTek, United States). The assessment was done in three biological replicates. 

***Total anti-oxidant capacity assay***

The cells were cultured in 6 cm plates until their confluence reached 70%. Then, they went under the treatments for 24 hr. The cells were washed, trypsinized, centrifuged, and suspended in phosphate-buffered saline (PBS) to obtain a cell concentration of approximately 1 million/ml. The cells were ruptured via freeze-thaw cycles to bring out their components. Next, they were centrifuged at 3000 RPM for 20 min. The supernatants were collected and further steps were performed according to the manufacturer’s manual. Finally, the absorbance was read at 412 nm using Synergy™ HTX Multi-Mode Microplate Reader (BioTek, United States). The assessments were done in three biological replicates. 

***Quantitative PCR assay***

The cells were cultured in 6 cm plates until their confluence reached 70%. Then, they went under the treatments for 24 hr. After that, the cells were washed, trypsinized, centrifuged, and their total RNA was extracted instantly using the EZ-10 DNAaway RNA extraction kit (Biobasics, Canada). Briefly, the cells were ruptured and homogenized by vortexing and the extraction steps were performed according to the manufacturer’s manual. The concentration and purity of the RNA samples were assessed by reading their absorbance at 230, 260, and 280 nm. Then, the Reverse transcription step was performed using the cDNA synthesis kit according to the manufacturer’s instructions (Favorgen, Taiwan). 

Real-time quantitative PCR was performed using the Corbett Rotor-Gene 6000 Real-Time PCR apparatus (Corbett Research, Australia) and SYBR Premix Ex Taq (Tli. RNase H Plus) (Takara, Japan). Briefly, the synthesized cDNA (1 μl), forward and reverse primers (1 μl of each at 10 pM), 10 μl of the 2x-PCR master mix, and 7 μl of molecular water (Aminsan, Iran) were mixed in a 0.2 ml PCR tube for each reaction. The PCR program was set as follows: an initial denaturation step (95 °C for 5 min); 40 cycles of amplification (12 sec at 95 °C, 15 sec at 60 °C, 20 sec at 72 °C); and a final extension step at 72 °C for 5 min. The expression levels of the genes were calculated based on the ΔΔCT method using LinRegPCR software version 2017.0. To confirm the specific amplification of the desired PCR products, melting curve analysis was used. The assessments were done in three biological replicates. The primer sequences are displayed in Table 1. 

***Apoptosis assay***

The cells were cultured in T-25 flasks until their confluence reached 70%. Then, they went under the treatments for 24 hr. Then, the apoptosis level of the cells was assessed using BD FACS Calibur (BD biosciences, USA). Phosphatidyl Serine Detection Kit. Briefly, the cells were washed, trypsinized, and centrifuged. After that, 500 μl 1X binding buffer was added to each tube. Next, 5 μl 7AAD and 5 μl Annexin V-PE were added to the tubes and incubated for 15 min at 4 °C. Then, 1 ml 1X binding buffer was added to each tube and centrifuged for 5 min at 1500 rpm. Since 7AAD and Annexin V-PE had overlap in their absorbance, four extra tubes were used to correct and set the samples as follows: a tube without any dye (blank), a tube containing 7AAD, a tube containing Annexin V-PE, and a tube containing both 7AAD and Annexin V-PE. 

***Cell cycle assay***

The cells were cultured in T-25 flasks until their confluence reached 70%. Then, they went under the treatments for 24 hr. Then, the cells were washed, trypsinized, and centrifuged. Next, 50 μl cold PBS was added to each tube and gently vortexed. Next, 1 ml super-chilled ethanol 70% was added gradually, and gently vortexed again. After that, the cells were centrifuged for 5 min at 1500 rpm, the supernatant was discarded and the cells were washed with PBS and centrifuged again. For the next step, PI Master Mix (prepared out of 40 μl PI, 10 μl RNase, and 950 μl PBS) was added to each tube to obtain a cell suspension with a concentration of 5 × 10^5^ cells/ml. The cells were incubated for 30 min at room temperature. Finally, the samples were analyzed by the BD FACS Calibur system (BD biosciences, USA). 

***Data analysis***

For analyzing the data, SPSS software version 27 was recruited. Normal distribution and variance heterogeneity of the data were assessed with Leven’s test. ANOVA test (followed by Tukey’s test) was used to evaluate the significant differences between the experimental groups. *P*<0.05 was considered significant. 

## Results

**Figure 1:**
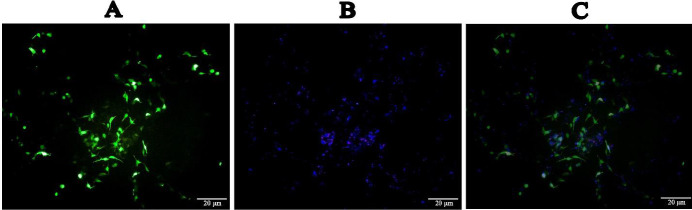
Microscopic figure of SH-SY5Y cells. A) The green cells are SH-SY5Y cells transfected by the pCDH vector and contain both the survivin gene and the green fluorescent protein (GFP) gene. B) SH-SY5Y cells stained with DApi stain. C) A merged figure combining Figures A and B

The transfection rate was approximately 50%, based on dividing the GFP-positive cells into all cells (Figure 1). 

The results of the ANOVA test for total anti-oxidant capacity (TAC) test (DF=20, F=403.44) showed that the rotenone group had a significantly lower anti-oxidant capacity compared with the healthy normal group (*P*<0.001). Over-expression of survivin could significantly increase the anti-oxidant capacity of cells exposed to rotenone (*P*<0.001) (Figure 3). Additionally, the anti-oxidant capacity of the group that over-expressed survivin (without rotenone treatment) was significantly higher than the healthy normal group (*P*<0.001). 

The results of the ANOVA test for gene expression analysis (DF=20, F=158.90) showed that the expression level of the survivin gene was increased significantly in the experimental groups, which over-expressed the survivin gene (*P*<0.001). So, the expression levels of survivin in these groups were approximately 140–160 folds higher than in the healthy normal group (Figure 4). 

The results of the ANOVA test for gene expression analysis showed that catalase was the only gene, which significantly decreased in the Rotenone group compared with the Healthy normal group (*P*=0.003) (DF=20, F=54.83). Over-expression of the survivin gene could prevent the decrease in catalase gene induced due to rotenone treatment (*P*=0.02). The genes bax (*P*<0.001) (DF=20, F=34.62), bcl2 (*P*=0.041) (DF=20, F=22.56), caspase 3 (*P*<0.001) (DF=20, F=104.05), caspase 8 (*P*<0.001) (DF=20, F=163.94), P53 (*P*<0.001) (DF=20, F=349.54), β-catenin (*P*=0.007) (DF=20, F=26.02), and ATG7 (*P*=0.044) (DF=20, F=5.85) were significantly increased in the Rotenone group compared with Healthy normal group. Over-expression of the survivin gene could prevent the increase in these genes, induced due to rotenone treatment, except β-catenin and ATG7. The genes SOD, caspase 9, SMAC, mTOR, AMPK, and RPS18 remained unchanged between the healthy normal group and the rotenone group. However, the groups, which over-expressed the survivin gene, had a significant increase in the expression level of the mTOR gene compared with the healthy normal group and rotenone group (*P*<0.001) (DF=20, F=240.40) (Figure 5). 

The results of cell death analysis by flow cytometry and annexin V-PE/7AAD showed that both early and late apoptosis was higher in the Rotenone group (43.20%) compared with the Healthy normal group (13.32%). However, over-expression of the survivin gene prevented the induction of apoptosis by rotenone (16.22%) (Figure 6). This experiment was performed once, due to limitations in funding and research facilities. 

The results of cell cycle analysis with flow cytometry & propidium iodide showed that the proportion of G2/G1 was higher in the rotenone group (64.98%) compared with the Healthy normal group (24.30%). Over-expression of the survivin gene prevented the increase of the proportion and decreased the frequency of the G2 step in cells. (33.37%) (Figure 7). This experiment was performed once, due to limitations in funding and research facilities. 

**Table 1:** The sequences of primer sets used to amplify the genes in this study

#	Gene	Forward primer (Tm) 5’→3’	Reverse primer (Tm) 5’→3’	Product length
1	AMPK	CCCTTCCATTTGATGATGACC (55.9)	CCCTGATATCTTTGATTGTGG (55.4)	159
2	ATG7	GTTGGATGGGAAAAGAACCAG (56.8)	TGTCCAAGTCTAAAGTAGGAAC (54.7)	151
3	BAX	TTTTGCTTCAGGGTTTCATCC (55.6)	TGCAGCTCCATGTTACTGTC (56.5)	157
4	BCL2	ATGCATTGTCAGTGATGTACC (56.6)	TTCCCTTTGGCAGTAAATAGC (56.2)	157
5	Caspase3	CAAAGATCATACATGGAAGCG (54.6)	GCTGCATCGACATCTGTAC (56)	163
6	Caspase8	AGACTCCAGGAAAAGAGAATG (56.6)	AGTTCCCTTTCCATCTCCTC (57.6)	131
7	Caspase9	CCAGAGATTCGCAAACCAG (55.8)	TTGTTGATAATGAGGCAGTGG (54.9)	167
8	Catalase	CAAGCTGGTTAATGCAAATGG (54.9)	GGAGGGGTACTTTCCTGTG (54.3)	169
9	CateninB	CTCATCCCACTAATGTCCAG (55.2)	CAGCCTTATTAACCACCACC (56.3)	165
10	GAPDH	GACATCAAGAAGGTGGTGAAG (56.6)	CTTGACAAAGTGGTCGTTGAG (54.6)	159
11	RPS18	GTGGGCCGAAGATATGCTC (58.1)	ATCTTGTACTGGCGTGGATTC (59.3)	131
12	mTOR	AACCATGTCCTAAGCTGTG (56.8)	CTTATGGGCGAAGTCCTTTG (55.2)	162
13	P53	ACTGACATTCTCCACTTCTTG (57.0)	CGGGACAAAGCAAATGGAAG (57.6)	147
14	SMAC	GACGATTGGCTTTGGAGTAAC (56.9)	GAGAGAGAAAGGTAGAGGTGC (57.3)	134
15	SOD1	CAGGTCCTCACTTTAATCCTC (55.4)	AATGATGCAATGGTCTCCTG (56.4)	161
16	Survivin	CACCGCATCTCTACATTCAAG (56.2)	GTCTGGCTCGTTCTCAGTG (55)	111
17	Vector	GGATAGCGGTTTGACTCACG (58.7)	CCAGCTCCTTGAAGCAGAAG (58.5)	451

**Table 2:** Results of Leven's test to assess the homogeneity of variances in statistical assessments

***SOD1: *** p = 0.872	***Caspase9:*** p = 0.499	***AMPK:*** p = 0.482
***Catalase:*** p = 0.641	***P53:*** p = 0.604	***ATG7:*** p = 0.548
***BAX:*** p = 0.381	***SMAC:*** p = 0.549	***RPS18:*** p = 0.378
***BCL-2: *** p = 0.525	***CateninB:*** p = 0.305	***MTT assay:*** p = 0.615
***Caspase3:*** p = 0.517	***Survivin:*** p = 0.385	***TAC assay:*** p = 0.918
***Caspase8:*** p = 0.481	***mTOR:*** p = 0.273	---

* *P*<0.05 indicated non-normal distribution and lack of homogeneity of variances

**Figure 2:**
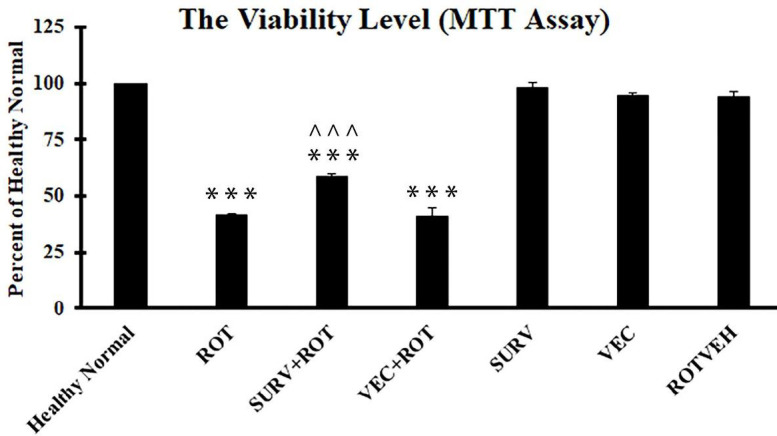
Viability of the experimental groups of SH-SY5Y cell line. Data were expressed as a percent of the control group (Healthy normal group). Data are presented as mean ± standard deviation (n=3 biological replicates). ANOVA test was recruited. *P*-value<0.05 was considered significant. ***P-value at <0.001 represents a significant difference with the healthy normal group. ^^^*P*-value at <0.001 represents a significant difference with the Rotenone group

ROT: rotenone; SURV: survivin; VEC: null vector; ROTVEH: rotenone vehicle

**Figure 3:**
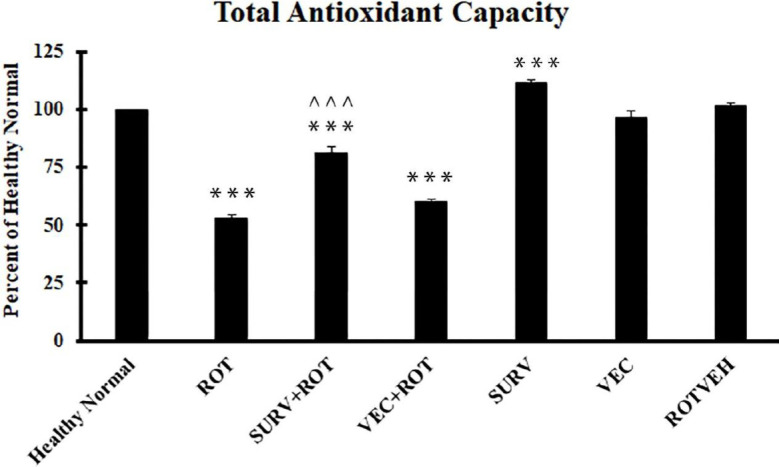
Total anti-oxidant capacity of the experimental groups of SH-SY5Y cell line. Data were expressed as a percent of the control group (Healthy normal group). Data are presented as mean ± standard deviation (n=3 biological replicates). ANOVA test was recruited. *P*-value<0.05 was considered significant. ***P-value at <0.001 represents a significant difference with the healthy normal group. ^^^*P*-value at <0.001 represents a significant difference with the Rotenone group

ROT: rotenone; SURV: survivin; VEC: null vector; ROTVEH: rotenone vehicle

**Figure 4:**
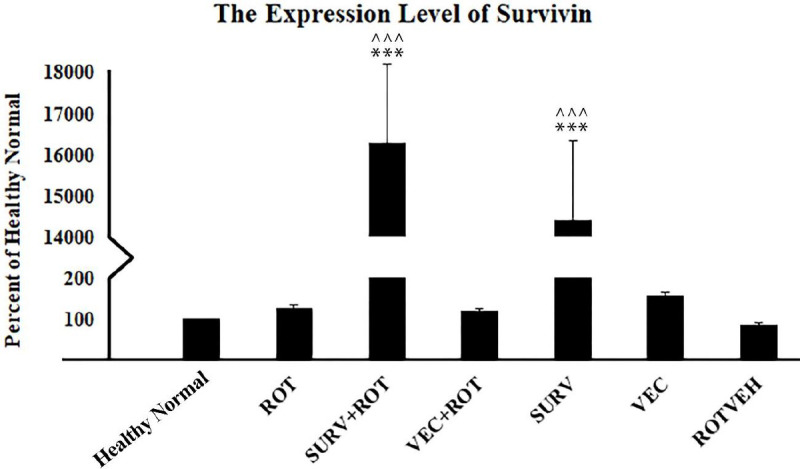
Expression level of survivin gene in the experimental groups of SH-SY5Y cell line. The results were normalized to GAPDH and the data were expressed as a percent of the control group (Healthy normal group). Data are presented as mean ± standard deviation (n=3 biological replicates). ANOVA test was recruited. *P*-value<0.05 was considered significant. *P-values at<0.05, **P-values at <0.01, and ***P-values at <0.001 represent significant differences with healthy normal group. ^*P*-values at <0.05, ^^*P*-values at <0.01, and ^^^*P*-values at <0.001 represent significant differences with Rotenone group

ROT: rotenone; SURV: survivin; VEC: null vector; ROTVEH: rotenone vehicle

**Figure 5:**
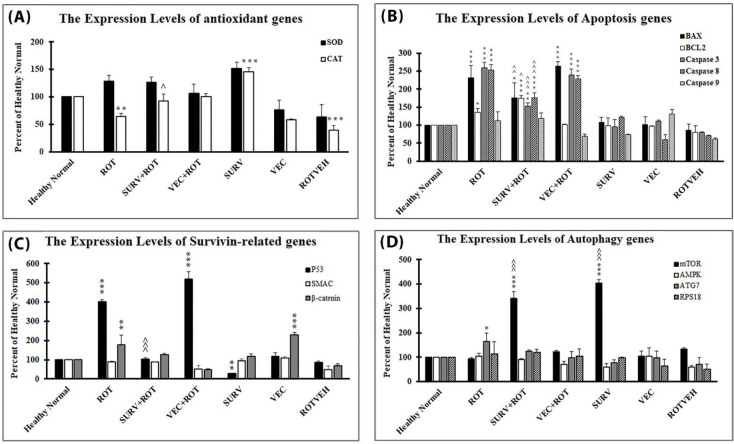
Expression levels of (A) anti-oxidant genes, (B) apoptosis genes, (C) survivin-related genes, and (D) autophagy genes in the experimental groups of the SH-SY5Y cell line. The results were normalized to GAPDH and the data were expressed as a percent of the control group (Healthy normal group). Data are presented as mean ± standard deviation (n=3 biological replicates). ANOVA test was recruited. *P*-value<0.05 was considered significant. *P-values at <0.05, **P-values at <0.01, and ***P-values at <0.001 represent significant differences with healthy normal group. ^*P*-values at <0.05, ^^*P*-values at <0.01, and ^^^*P*-values at <0.001 represent significant differences with Rotenone group

ROT: rotenone; SURV: survivin; VEC: null vector; ROTVEH: rotenone vehicle

**Figure 6:**
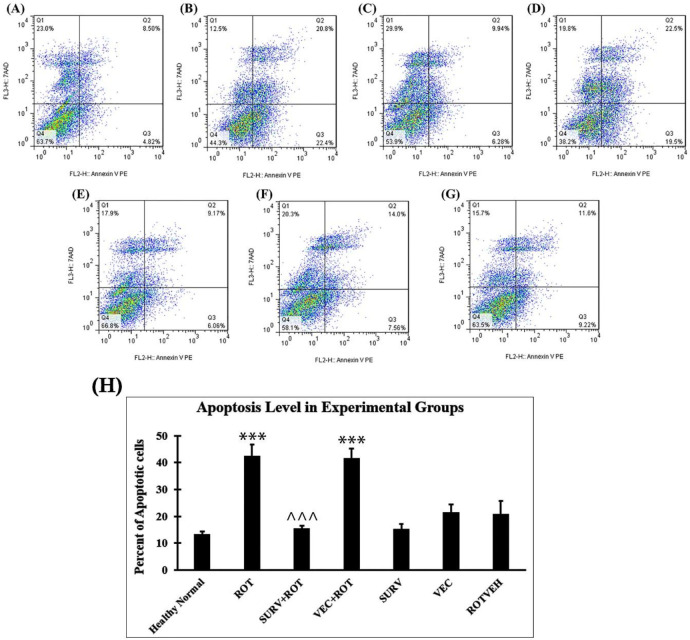
Assessment of cell death by Annexin V-PE binding and 7AAD uptake in contour plot of flow cytometry. The experimental groups are presented according to the order mentioned earlier in Methods section 2.4: (A) Healthy normal group, (B) Rotenone group, (C) Rotenone+Survivin, (D) Rotenone+Null vector, (E) Survivin, (F) Null vector, (G) Rotenone vehicle. Q1:Necrosis, Q2: Late Apoptosis, Q3: Early Apoptosis, Q4: Live Cells. (H) Apoptosis levels in the experimental groups of SH-SY5Y cell line. Data were expressed as the percent of apoptotic cells. Data are presented as mean ± standard deviation (n=3 biological replicates). ANOVA test was recruited. *P*-value < 0.05 was considered as significant. ***P-value at <0.001 represents a significant difference with the healthy normal group. ^^^*P*-value at <0.001 represents a significant difference with the Rotenone group

ROT: rotenone; SURV: survivin; VEC: null vector; ROTVEH: rotenone vehicle

**Figure 7:**
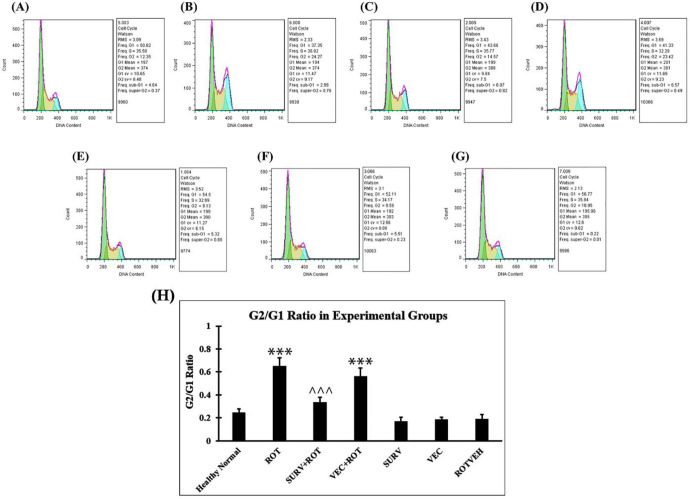
Cell cycle analysis with flow cytometry & propidium iodide. The experimental groups are presented according to the order mentioned earlier in the Methods section (2.4): (A) Healthy normal group, (B) Rotenone group, (C) Rotenone+Survivin, (D) Rotenone+Null vector, (E) Survivin, (F) Null vector, (G) Rotenone vehicle. (H) G2/G1 ratio in the experimental groups of SH-SY5Y cell line. Data were expressed as the percent of apoptotic cells. Data are presented as mean ± standard deviation (n=3 biological replicates). ANOVA test was recruited. *P*-value<0.05 was considered significant. ***P-value at <0.001 represents a significant difference with the healthy normal group. ^^^*P*-value at <0.001 represents a significant difference with the Rotenone group ROT: rotenone; SURV: survivin; VEC: null vector; ROTVEH: rotenone vehicle

## Discussion

The results of the current study showed that over-expression of survivin could increase the viability of SH-SY5Y cells treated with rotenone. Meanwhile, over-expression of survivin could prevent loss of total anti-oxidant capacity in the cells treated with rotenone. Accordingly, it can be hypothesized that over-expression of survivin implies a part of its protective effect via increasing the anti-oxidant capacity of the cells. Consistently, we could observe that over-expression of survivin could significantly increase the expression level of the catalase gene, as an important constituent of the endogenous anti-oxidant system of human cells. In this regard, Baratchi *et al*. (2011) showed that survivin could activate the ROS scavengers, including catalase, in the SK-N-SH cell line (14). Additionally, Kwee *et al*. (2008) proved a correlation between intracellular ROS level, apoptosis rate, and the expression level of survivin in K562 cells. They stated that the expression level of survivin was closely related to the ROS concentration in the cells (17). According to the results of the current study and the other studies mentioned here, it can be concluded that survivin may play an important role in regulation of endogenous anti-oxidant system of the cells. 

However, we assume that the protective effects of over-expression of survivin were not solely dependent on increasing the anti-oxidant capacity of the cells. As shown in the results, survivin had significant impacts on the apoptosis-related genes. For example, survivin could prevent the increase in Bax/Bcl2 ratio due to rotenone toxic effect. The increased Bax/Bcl2 ratio may increase the susceptibility to apoptosis. Consistently, the results of the apoptosis assay confirmed that over-expression of survivin could decrease both early and late apoptosis in rotenone-treated SH-SY5Y cells. As another important point, the results of gene expression analyses showed that the expression levels of caspase 3 and caspase 8 increased significantly in SH-SY5Y cells due to rotenone treatment, while the expression levels of caspase 9 remained unchanged. Over-expression of survivin could also decrease the expression levels of both caspase 3 and caspase 8. These results indicated that apoptosis was probably conducted via the extrinsic pathway (since the extrinsic pathway is conducted via caspase 8 and the intrinsic pathway is conducted via caspase 9). The results also confirmed that over-expression of survivin probably could exert an effect on caspase 3 and caspase 8 to prevent apoptosis. In this regard, Tang *et al*. (2009) proved the inhibitory effect of survivin on caspases, especially caspase 3 (18). In addition, researchers (2020) mentioned a correlation between down-regulation of survivin and increase in caspase 8 and 3 in two human leukemia cell lines (K562 and KG1a) (19). These results and literature review, all together, suggest that survivin has a pervasive effect on various constituents of the apoptosis pathway and may play a key role in determining the cellular fate. 

The results of the current study also showed that the expression level of P53 increased significantly in SH-SY5Y cells due to rotenone treatment and over-expression of survivin prevented the increase of P53 in the cells. P53 is a central protein in controlling the cell cycle. Thus, it seems that the increase in G2/G1 ratio in SH-SY5Y cells, due to rotenone treatment, is at least partially dependent on this increase in P53. In this regard, Wang *et al*. (2020) reported that rotenone could activate P53 in SH-SY5Y cells and increase the expression level of Bcl-2 via up-regulating Bcl-2-binding component 3 (also known as PUMA) (20). It is noteworthy that we could also observe a significant increase in the expression level of the Bcl-2 gene in the rotenone treated group. Additionally, consistent with our results, previous studies indicated that P53 could arrest the cell cycle at the G2 stage via direct/indirect interactions with various sets of proteins including CDC2, RPRM, B99, and MCG10 (21, 22). However, an increase in P53 may also arrest the cells in the G1 stage and this was not consistent with our results. To explain this result, the cells arrested in G1 by P53 may have two fates: they repair their damages and restart the cell cycle or their damages are too much to withstand and they undergo the apoptosis process (23). We hypothesize that a considerable portion of the cells arrested in G1 may undergo apoptosis and this is why our results indicated that G2/G1 ratio was high in the rotenone treated group. 

The results of the current study showed that the expression level of β-catenin increased due to rotenone treatment. However, over-expression of survivin could not significantly decrease this rotenone-induced up-regulation. It is noteworthy that the results of this study were only limited to the assessment of transcription levels. Thus, it is not inconceivable if we hypothesize that survivin might have a greater and more significant effect on β-catenin if we had assessed their correlation with the western blot technique or functional analyses. Nevertheless, this hypothesis needs further investigation. In this regard, Marchetti (2018) stated that an increase in the expression level of β-catenin may be protective against oxidative stress and dopaminergic cell health during PD (24). Accordingly, we hypothesize that the increase in the expression levels of β-catenin in rotenone-treated SH-SY5Y cells in our study might be due to a compensatory mechanism to withstand the toxic effects of rotenone. However, this needs further investigation. 

Regarding the autophagy-related genes, our results did not show a significant increase in the expression level of the mTOR gene due to rotenone treatment. However, the two survivin over-expressing groups showed approximately four-fold increases in the expression levels of the mTOR gene. Indeed, the expression level of the mTOR gene was affected by survivin more than all of the studied genes. Accordingly, it can be assumed that survivin may apply a part of its protective effect via the mTOR gene. In this regard, many previous studies reported the inhibitory effect of mTOR on the expression level of the survivin gene (25, 26). However, to the best of our knowledge, the current study was the first report on the contrariwise effect of survivin on the expression level of mTOR. Taken together, these results emphasize the probable role of these two genes and their cooperation in regulating and conducting apoptosis and autophagy processes. Therefore, these genes seem interesting targets for designing research studies to develop novel gene therapy-based medical approaches. 

The results of the current study showed that the expression level of ATG-7 increased due to rotenone treatment. However, over-expression of survivin could prevent the significant increase. In this regard, Lin *et al*. (2020) reported an inverse correlation between the expression levels of survivin and ATG-7 genes (27). Our study failed to observe the same correlation. However, our results indicated the importance of ATG-7 in the development of a rotenone-induced *in vitro* PD model. 

It is important to mention that in this study the efficiency of cellular transfection was only 50%. Therefore, we assume that the effect of survivin over-expression on the studied factors is probably even greater than it was observed. It is also noteworthy that in the current study, only the transcription levels of the studied genes were assessed. The expression of the genes at the translation level and the effect of survivin on their activation were not assessed. Thus, further investigations (protein quantifications and functional analyses) are needed to evaluate the probable effects of survivin on its other probable targets. 

It is important to mention that the current study was only an *in vitro* study and its finding has still a long way ahead to be validated and translated into clinical use. However, as discussed in the introduction, developing novel therapeutic approaches have great importance for PD. The current study may be a basis for future *in vivo* and clinical studies. It is very important to maintain the valuable remaining dopaminergic neurons in PD patients, to prevent the progression of the disease. Therefore, designing novel therapeutic methods with the use of genes like survivin, which can inhibit apoptosis and oxidative stress (two important processes participating in the development and progression of PD), may become promising clinical approaches. However, it is important to keep in mind that gene therapy-based methods are relatively novel complicated methods, which are like a double-edged sword and need hierarchical step-by-step studies, and vast investigations, optimizations,and considerations are needed in every pre-clinical and clinical step. 

## Conclusion

The results of the current study showed that over-expression of survivin could prevent the loss of SH-SY5Y cells during the development of the *in vitro* PD model by rotenone. Survivin implied this effect by regulating the genes in key cellular processes, including anti-oxidation, apoptosis, cell cycle, and autophagy. 

## Authors’ Contributions

AR Provided conceptualization, investigation, project management, methodology, data analysis, and writing the original draft; FF and BN Supervised, reviewed, and edited the manuscript; FS Helped investigate, supervised, reviewed, and edited the manuscript; MK Conceived the study, investigated, managed the project, provided methodology, supervised, reviewed, and edited the manuscript. The authors declare that all data were generated in-house and that no paper mill was used. 

## Data Availability

Data are available on request from the authors. 

## Compliance with Ethical Standards

This article does not contain any studies with human participants or animals performed by any of the authors. 

## Conflicts of Interest

All authors confirm that they have no conflicts of interest.
